# Inner ear dysfunction in caspase-3 deficient mice

**DOI:** 10.1186/1471-2202-12-102

**Published:** 2011-10-12

**Authors:** Tomoko Makishima, Lara Hochman, Patrick Armstrong, Eric Rosenberger, Ryan Ridley, Minna Woo, Adrian Perachio, Scott Wood

**Affiliations:** 1 Department of Otolaryngology, University of Texas Medical Branch, Galveston, Texas, USA; 2 School of Medicine, University of Texas Medical Branch, Galveston, Texas, USA; 3 Department of Medicine, University of Toronto, Ontario Cancer Institute, Toronto, Canada; 4 Universities Space Research Association, NASA Johnson Space Center, Houston, Texas, USA

## Abstract

**Background:**

Caspase-3 is one of the most downstream enzymes activated in the apoptotic pathway. In caspase-3 deficient mice, loss of cochlear hair cells and spiral ganglion cells coincide closely with hearing loss. In contrast with the auditory system, details of the vestibular phenotype have not been characterized. Here we report the vestibular phenotype and inner ear anatomy in the caspase-3 deficient (*Casp3*^*-/-*^) mouse strain.

**Results:**

Average ABR thresholds of *Casp3*^*-/- *^mice were significantly elevated (*P *< 0.05) compared to *Casp3*^*+/- *^mice and *Casp3*^*+/+ *^mice at 3 months of age. In DPOAE testing, distortion product 2F1-F2 was significantly decreased (*P *< 0.05) in *Casp3*^*-/- *^mice, whereas *Casp3*^*+/- *^and *Casp3*^*+/+ *^mice showed normal and comparable values to each other. *Casp3*^*-/- *^mice were hyperactive and exhibited circling behavior when excited. In lateral canal VOR testing, *Casp3*^*-/- *^mice had minimal response to any of the stimuli tested, whereas *Casp3*^*+/- *^mice had an intermediate response compared to *Casp3*^*+/+ *^mice. Inner ear anatomical and histological analysis revealed gross hypomorphism of the vestibular organs, in which the main site was the anterior semicircular canal. Hair cell numbers in the anterior- and lateral crista, and utricle were significantly smaller in *Casp3*^*-/- *^mice whereas the *Casp3*^*+/- *^and *Casp3*^*+/+ *^mice had normal hair cell numbers.

**Conclusions:**

These results indicate that caspase-3 is essential for correct functioning of the cochlea as well as normal development and function of the vestibule.

## Background

Caspase-3 is one of the most commonly shared downstream executioners in different apoptotic pathways. In the inner ear, apoptosis and molecules involved in the apoptosis pathways play an important role during development [1,2] and in response to stress in adulthood. Caspase-3 activation is induced in response to ototoxic stress such as aminoglycoside antibiotics, cisplatin or noise, which in turn leads to hair cell death [3,4].

Mice deficient of molecules in the apoptotic pathway are useful tools to investigate their direct molecular role(s) in the inner ear. Caspase-3 deficient mice, although reported to have neuronal defects and a short life span, have no other obvious developmental abnormalities [5]. Back-crossing of the *Casp3 *mutant mice to the C57BL/6 strain, resulted in a longer lifespan without significant gross anatomical abnormalities [6-8]. These mice were found to have a smaller body size and to exhibit decreased hearing, hyperactivity, and circling behavior, suggestive of inner ear dysfunction.

Detailed characterization of the auditory phenotypes of two strains of caspase-3 deficient mice with a targeted deletion of exon 5-6, which encodes the QACRG pentapeptide motif in the catalytic domain of caspase-3, have been reported [7,8]. In these mice, although the development of the organ of Corti seems normal, degeneration of the auditory hair cells and spiral ganglion cells [7], as well as hyperplasia of supporting cells (border cells) were observed [8] coinciding with severe progressive hearing loss. In addition, *Melody*, an ENU mutant with a point mutation within the catalytic domain of *Casp3*, has been shown to have similar auditory phenotype. These mice also display severe hearing loss as well as loss of auditory hair cells and spiral ganglion cells [9].

In contrast with the detailed characterization of the auditory phenotype in these *Casp3 *mutant mice, details of the vestibular phenotype have not been reported. Here we report the characterization of the vestibular phenotype in the caspase-3 deficient mouse.

## Results

### Profound hearing loss in caspase-3 deficient mice

**Figure 1:**
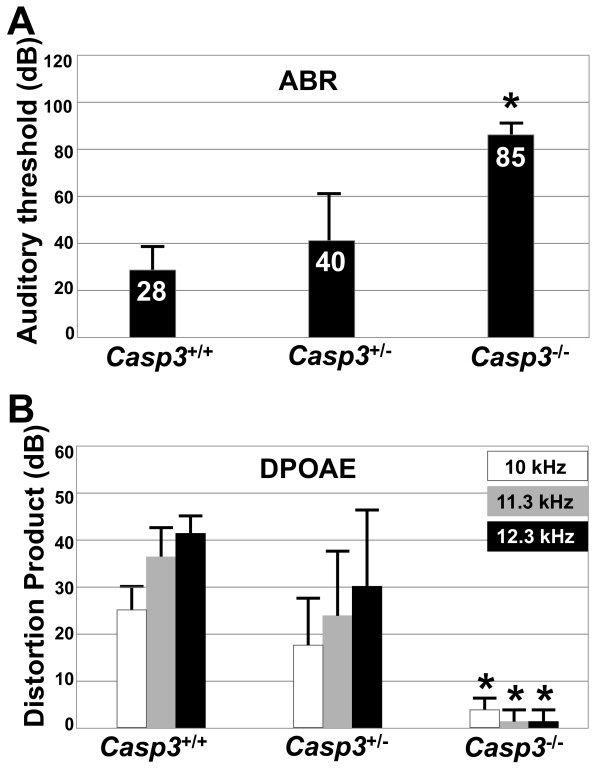
**Auditory dysfunction in caspase-3 deficient mice**. (A) Auditory brainstem response (ABR) thresholds to click stimuli at 3 months of age. *Casp3*^-/- ^(*n *= 6) mice had a significantly higher threshold (*, *P *< 0.05) compared to *Casp3*^+/- ^(*n *= 13) and *Casp3*^+/+ ^(*n *= 5) mice. (B) Distortion product otoacoustic emission (DPOAE). Averages of distortion product 2F1-F2 at F2 frequencies of 10 kHz, 11.3 kHz, and 12.3 kHz were compared at 3 months of age. *Casp3*^-/- ^(*n *= 5) mice had a significantly smaller distortion product (*, *P *< 0.05) compared to *Casp3*^+/- ^(*n *= 9) and *Casp3*^+/+ ^(*n *= 5) mice.

The auditory function was assessed by auditory brainstem response (ABR) and distortion product otoacoustic emission (DPOAE) at 3 months of age. ABR was used to objectively assess the function of the entire auditory pathway, whereas DPOAE evaluated the cochlear function. Average ABR thresholds were significantly elevated (*P *< 0.05) in *Casp3*^-/- ^mice (*n *= 6) at 85 ± 7 dB SPL, compared to heterozygous *Casp3*^+/- ^mice (*n *= 13) at 40 ± 22 dB SPL and wild type *Casp3*^+/+ ^mice (*n *= 5) at 28 ± 11 dB SPL (Figure 1A). In DPOAE testing, the distortion product 2F1-F2 was significantly decreased (*P *< 0.05) in *Casp3*^-/- ^mice (*n *= 5) compared to *Casp3*^+/- ^mice (*n *= 9) and *Casp3*^+/+ ^mice (*n *= 5), which showed comparable distortion products to each other (Figure 1B).

### Vestibular dysfunction in caspase-3 deficient mice

**Figure 2:**
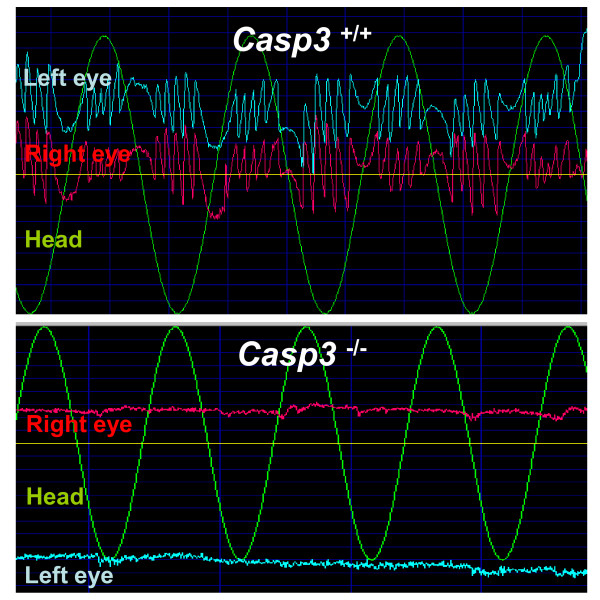
**Caspase-3 deficient mice have vestibular dysfunction**. Representative raw tracings of head velocity (green line), left horizontal eye movement (light blue line), and right horizontal eye movement (red line) at 0.2 Hz, 90°/sec sinusoidal rotation. *Casp3*^-/- ^mice (lower panel) showed no ocular response to the stimulus, whereas *Casp3*^+/+ ^mice (upper panel) showed robust response.

**Figure 3:**
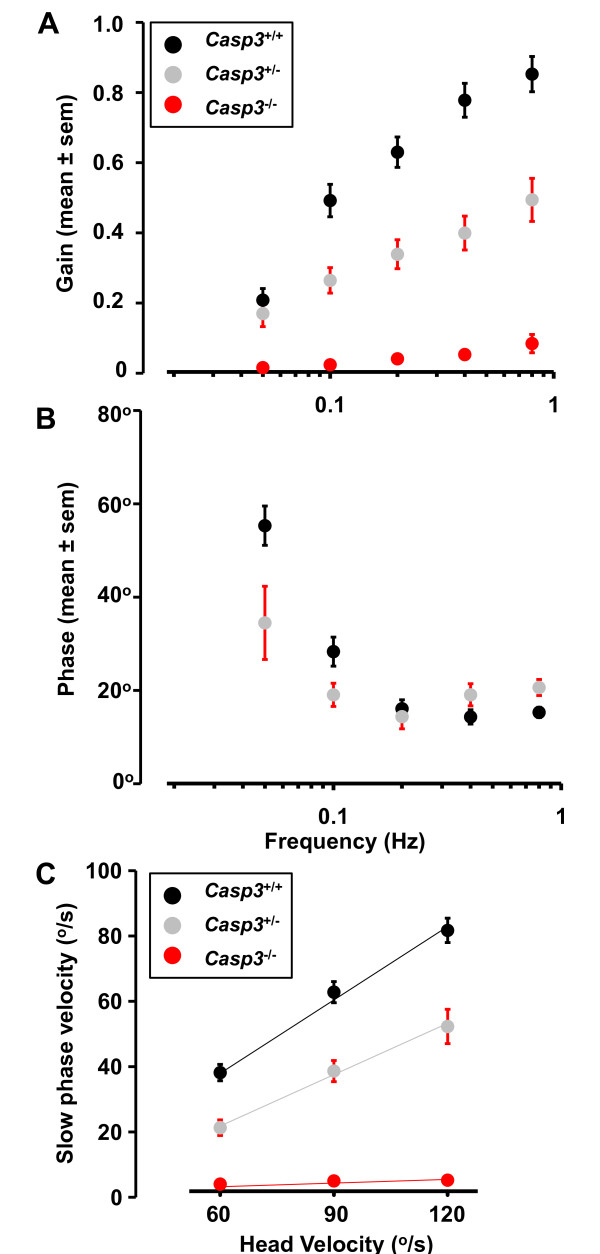
**Vestibular ocular reflex (VOR) in caspase-3 deficient mice**. VOR in darkness. Gain (A) and phase (B) at different frequencies were compared among all genotypes. Negligible VOR responses were recorded for *Casp3*^*-/- *^mice at all stimulus conditions. Heterozygous *Casp3*^*+/- *^mice performed at an intermediate level compared to wild type *Casp3*^*+/+ *^mice, with VOR impairment at higher frequencies. (C) VOR linearity at 0.2Hz. The sensitivity (increase in SPV for increase in head velocity) was reduced for the heterozygous *Casp3*^*+/- *^group, and negligible for the knockout *Casp3*^*-/- *^group. Black circle, *Casp3*^*+/+*^. Gray circle, *Casp3*^*+/-*^. Red circle, *Casp3*^*-/-*^. SPV, slow phase velocity.

Most *Casp3*^-/- ^mice were hyperactive and exhibited circling behavior with a tendency towards a unilateral directional rotation when excited. We observed that about 70% of the mice circle counter-clockwise (left direction), while 25% circle clockwise (right direction). We tested the horizontal vestibulo-ocular reflex (VOR) in the dark using near-infrared video-oculography to assess vestibular function in different genotypes. The frequency response of the VOR in darkness for each group was characterized during horizontal rotation at 5 frequencies ranging from 0.05Hz to 0.8Hz, with peak velocity of 60 degrees/sec. Little response at any frequency or velocity was recorded for *Casp3*^-/- ^mice (*n *= 10) (Figure 2, 3). Both heterozygous *Casp3*^+/- ^(*n *= 13) and wild type *Casp3*^+/+ ^(*n *= 14) mice exhibited a high-pass filtered VOR response, with higher gains and lower phase errors with increasing stimulus frequency (Figure 3A, B). The VOR at 0.2 Hz was also linear over the range of peak velocities from 60 to120 deg/sec for both groups (Figure 3C). The gain of the *Casp3*^+/- ^mice was intermediate between *Casp3*^-/- ^and *Casp3*^+/+ ^mice (*P *< 0.05).

### Inner ear dysmorphism in caspase-3 deficient mice

**Figure 4:**
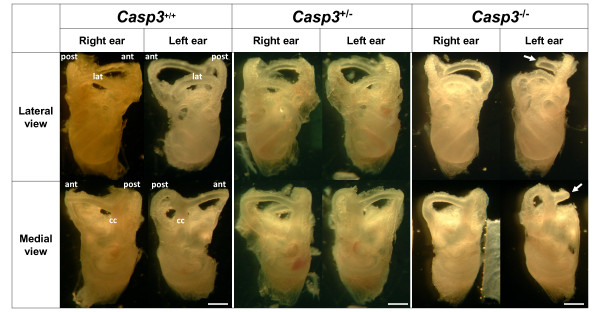
**Vestibular dysmorphism in caspase-3 deficient mice**. Temporal bones of *Casp3*^-/-^, *Casp3*^+/- ^and *Casp3*^+/+ ^mice at postnatal day 20. Truncation of the left superior semicircular canal in a *Casp3*^-/- ^mouse is shown (arrow). Most *Casp3*^-/- ^(*n *= 14) had variable degrees of dysmorphisms in the anterior semicircular canal, whereas all *Casp3*^+/- ^(*n *= 20) and *Casp3*^+/+ ^(*n *= 14) had normal inner ear morphology. ant, anterior semicircular canal. lat, lateral semicircular canal. post, posterior semicircular canal. cc, common crus. Scale bar, 1mm.

Anatomical analysis of the inner ear of *Casp3*^-/- ^mice revealed gross hypomorphology in the vestibule, mainly in the anterior semicircular canal (Figure 4). Various degrees of malformations in the anterior semicircular canal were observed in 14 out of 15 *Casp3*^-/- ^mice tested, whereas all of the *Casp3*^+/- ^mice (*n *= 20) or *Casp3*^+/+ ^(*n *= 14) had normal gross semicircular canal anatomy. The most common malformation was a decreased arc size of the anterior semicircular canal (*n *= 10). Other severe malformations included truncation or aplasia of the anterior semicircular canal. Those with severe anterior semicircular canal malformations were often accompanied by a hypomorphic lateral semicircular canal (*n *= 3), but to a much smaller frequency. However, the posterior semicircular canal was always preserved. Most malformations were observed unilaterally in the left ear (*n *= 6) or in the right ear (*n *= 3), but also in bilateral ears (*n *= 5). Gross anatomy of the cochlea appeared normal in all genotypes (Figure 4).

**Figure 5:**
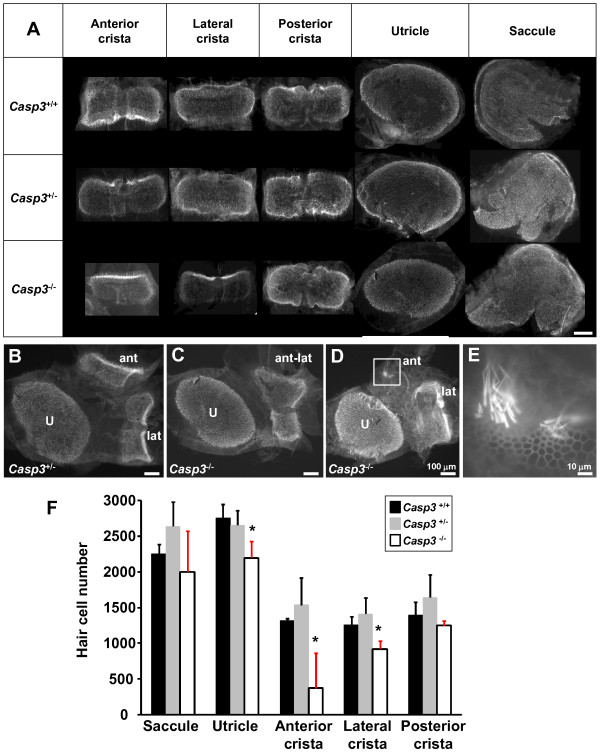
**Anterior semicircular canal dysmorphism in *Casp3***^***-/-***^**mice**. (A) Whole mount vestibular epithelia of *Casp3*^-/-^, *Casp3*^+/-^, and *Casp3*^+/+ ^mice. anterior- and lateral crista, and utricle were smaller, whereas the posterior crista and saccule were normal in size in *Casp3*^-/- ^mice. All vestibular sensory epithelia were normal in size and shape in *Casp3*^+/- ^and *Casp3*^+/+ ^mice. (B, C, D, E) Comparison of morphologic changes in utricle, anterior crista, and lateral crista. Examples of severe dysmorphisms in *Casp3*^-/- ^mice are shown in C and D, compared to normal utricle, anterior crista, and lateral crista in *Casp3*^+/- ^mice in B. (C) Anterior- and lateral crista was fused in some *Casp3*^-/- ^mice. (D) Significantly smaller size in anterior crista (white box) in *Casp3*^-/- ^mice. Although hair cell number in the anterior crista is significantly reduced, the morphology of the stereocilia of the cells seem normal in shape and size (E). A magnified image of the anterior crista (white box in D) is shown in E. (F) Hair cell numbers in the vestibular sensory epithelia. Average hair cell numbers ± S.D. of *Casp3*^+/+ ^(*n *= 3), *Casp3*^+/- ^(*n *= 5), and *Casp3*^-/- ^(*n *= 7) were compared. Black, *Casp3*^+/+^. Gray, *Casp3*^+/-^. White, *Casp3*^-/-^. Statistically significant differences (ANOVA, *P *< 0.05) are marked with *. U, utricle. ant, anterior crista. lat, lateral crista. post, posterior crista. Scale bar, 100 μm, except for in panel E (= 10 μ m).

Histological studies of sensory epithelia were consistent with gross anatomy findings. The crista of the anterior- and lateral semicircular canals was hypomorphic, whereas that of the posterior semicircular canal developed normally (Figure 5). Hair cell numbers were significantly smaller (*P *< 0.05) in the anterior- and lateral crista, and in the utricle, in *Casp3*^-/- ^mice (*n *= 7), whereas *Casp3*^+/- ^mice (*n *= 5) and *Casp3*^+/+ ^mice (*n *= 3) had normal hair cell numbers in all vestibular sensory epithelia (Figure 5F). While the size of the hair cells looked normal, the decreased number of hair cells seemed to be the main reason for the hypomorphic anterior crista ampullaris in *Casp3*^-/- ^mice (Figure 5E). In some *Casp3*^-/- ^mice, the anterior crista was absent, or fused with the lateral crista (Figure 5C). All *Casp3*^+/- ^mice and *Casp3*^+/+ ^mice observed to date had normal morphology and hair cell numbers in the vestibular sensory epithelia. Although most *Casp3*^-/- ^mice seemed to circle toward the more severely affected side of the ear, there was no significant difference between the hair cell number in the left ear and right ear (Student's *t *test, *P *> 0.05).

## Discussion

### Caspase-3 is essential for the maintenance of normal hearing

When different regions are targeted to delete a gene of interest, the knockout mice may exhibit different phenotypes depending on the background strain, even though the same gene is deleted [10]. The auditory function test results from ABR and DPOAE of our *Casp3 *^-/- ^mice were consistent with previously reported profound hearing loss in two different strains of *Casp3 *knockout mice [5,7]. The similar phenotype in the different mutant strains of *Casp3 *(Table 1) support a strong role for development and function in the inner ear. Taken together with the normal cochlear anatomy of the *Casp3*^-/- ^mice, these findings suggest an essential role of caspase-3 in the maintenance of normal hearing after the developmental stages of the cochlea.

**Table 1:** Comparison of *Casp3 *mutant mice

	*Casp3 *KO mice	*Casp3 *KO mice	*Melody *ENU mutant	***Casp3***^***ex3***^**KO mice**
	**(Takahashi et al., 2001**[7])	**(Morishita et al., 2001**[8])	**(Parker et al., 2010**[9])	(This study)
**Targeted deletion**	QACRG catalytic domain	QACRG catalytic domain	QACRG catalytic domain	Exon 3
**or mutation site**	Exons 5-6	Exons 5-6	C163S point mutation	
**Phenotype**	Survive on C57BL/6 background	Survive on C57BL/6 background	C3H background	Survive on C57BL/6 background
	Smaller body size	Smaller body size	Smaller body size	Smaller body size
	Progressive hearing loss	Progressive hearing loss	Hearing loss	Hearing loss
		Hyperactive		Hyperactive
		Circling behavior		Circling behavior
**Cochlear histology**	Hyperplasia of border cells	Loss of cochlear hair cells	Loss of cochlear hair cells	Loss of cochlear hair cells
	Degeneration of cochlear hair cells, spiral ganglion cells	Loss of spiral ganglion cells	Loss of spiral ganglion cells	

KO, knockout. ENU, N-ethyl-N-nitrosourea.

### Caspase-3 is important for development of the vestibule

Most mutant mice with inner ear phenotypes have extensive developmental defects in the vestibular organs [11]. The lateral semicircular canal is one of the most vulnerable, and morphologically affected [12]. On the other hand, those with either isolated anterior- or posterior semicircular canal defects are rare (Table 2). *Apaf1 *knockout mice and *Casp9 *knockout mice are a few of such mutants with specific anterior semicircular canal defect [13]. Interestingly, both apaf1 and caspase-9 are molecules involved in apoptosis. Apaf1 together with caspase-9, form a multiprotein complex called apoptosomes, which in turn activates downstream caspase-3, in the apoptotic pathway [14,15]. In these knockout mice, apoptosis is greatly reduced during development of the inner ear [13]. One possible mechanism leading to the phenotype is speculated to be the reduction of apoptosis, which in turn decrease the passive release of functional factors from the dying cells into the local environment [13]. Only a few genes are known to be exclusively expressed in a specific crista or canal during development [16]. There is no gene reported to be expressed specifically in the anterior crista or canal. Although many genes are ubiquitously expressed, differential expression of genes are required for the formation of each of the three cristae and canals[16]. This suggests an important role for this particular apoptotic cascade involving apaf1-caspase-9-caspase-3 in the development of the anterior semicircular canal.

**Table 2:** Mutant mice with semicircular canal dysmorphism

Mutant	Lateral SCC	Posterior SCC	Superior SCC	References
***Crsl***	+	nl	nl	[27]
***Ecl***	+	nl	nl	[28]
***Flouncer***	+	nl	nl	[29]
***Mt***	+	nl	nl	[30]
***Otx1***	+	nl	nl	[31]
***Prx1/2***	+	nl	nl	[32]
***Whl***	+	nl	nl	[33]
***Bcl2l***	nl	+	nl	[13]
***Apaf1***	nl	nl	+	[13]
***Casp9***	nl	nl	+	[13]
***Brn4/Pou3f4***	nl	nl	+	[34,35]
***Cdh7***	+	+	nl	[36]
***Edy***	+	+	nl	[30]
***Cyn***	+	+	nl	[30]
***Dz***	+	+	nl	[30]
***Lda***	+	+	nl	[30]
***Obt***	+	+	nl	[30]
***Todo***	+	+	nl	[30]
***Gbx2***	nl	+	+	[37]
***Dlx5***	+	+	+	[38]
***Fgf10***	+	+	+	[39]
***Hmx2***	+	+	+	[40]
***Hoxa1/Hoxb1***	+	+	+	[41]
***Netrin1***	+	+	+	[42]
***Nr4a3(Nor1)***	+	+	+	[43]

+, dysmorphic anatomy. nl, normal anatomy. SCC, semicircular canal.

Apoptosis plays an important role throughout the development of the inner ear [1], including the stages during the innervation of the vestibular epithelia from the sensory ganglion and the differentiation of the otic epithelia into the sensory epithelia [2]. In *Casp3*^-/- ^mice, in contrast to the normally developed cochlea, in the vestibule, the anterior crista, lateral crista, and utricle were hypomorphic at birth. The gross innervation to these three vestibular organs was reduced in our preliminary observation of the nerve fibers and vestibular ganglion cells. The anterior crista, lateral crista, and utricle are innervated by the superior vestibular nerve [17]. This may suggest that caspase-3 is one of the major apoptotic molecules involved in the development of the superior vestibular nerve, which innervates all three vestibular organs.

Either as a direct- or indirect consequence, caspase-3 seems to have an important role in the development of the vestibule, especially in the anterior- and lateral semicircular canals.

### Vestibular dysfunction in caspase-3 mutant mice

The horizontal VOR paradigm tested the vestibular ocular pathway for the lateral semicircular canal. The VOR results of the *Casp3*^+/+ ^mice were qualitatively similar to other studies in mice using both search coil [18] and videographic methods [19]. In our *Casp3 *^-/- ^mice, even those with relatively normal lateral semicircular canal morphology, had little response to any stimuli tested. Vidal et al. [20] showed that the mutant mice with deficient VOR function also had profound deficits in locomotion. It is not yet clear the extent to which peripheral versus central mechanisms contributes to the "circling behavior." The unidirectional circling behavior towards the more severely anomalous vestibule suggests that the vestibular system is functional to some degree postnatally, corresponding to the extent of hypomorphism of the vestibule. Further functional and histological studies on the inner ear and central vestibular pathways would be needed to clarify these questions.

Although *Casp3*^+/- ^did not exhibit any abnormal behavior or gross anatomical abnormalities, VOR analysis revealed an intermediate phenotype. Stimulation with rapid frequencies produced significant differences between *Casp3*^+/- ^and *Casp3*^+/+ ^mice, suggesting that adequate vestibular development has not occurred in *Casp3*^+/- ^mice.

In *Casp3*^+/- ^mice, we observed slightly increased hair cell numbers in most vestibular organs compared to *Casp3*^+/+ ^mice (Figure 5F). In mammals, regeneration has been observed in vestibular hair cells after damage from ototoxic agents [21-23]. Perhaps in the *Casp3 *mutants, caspase-3 insufficiency causes overall decrease in cell death at later stages of development or even after birth. One possible mechanism of mild vestibular dysfunction may be that perhaps in *Casp3*^+/- ^mice there is an imbalance in cell population and corresponding neurons which caused improper functional development.

In our studies, we used mice at 3 - 6 months of age, in order to avoid age related changes known to affect the C57BL6 mice background strain. In C57BL6 mice, VOR gain- and histological changes in the vestibular organ occur after six months of age [24]. Differences at the highest frequency in VOR performance, and whether there is a correlation with hair cell numbers in *Casp3*^+/- ^mice may be elucidated with further vestibular function analysis and histological studies in mice at different ages.

## Conclusions

The results in this study, together with previous inner ear studies on caspase-3 deficient mice, indicate that (1) caspase-3 is essential for normal function of the auditory and vestibular system, and (2) caspase-3 is important for the morphogenesis of the anterior semicircular canal. These mice can be useful tools for further studies, such as characterizing morphogenesis of the anterior semicircular canal, or as a control animal for developing vestibular function tests in mice.

## Methods

### Mice

Caspase-3 deficient mice were a gift from Minna Woo (University of Toronto). The generation of the *CPP32 *^ex3-/- ^( = *Casp3*^-/-^) are described [6]. The *Casp3 *gene was disrupted by targeted deletion of exon 3, which leads to termination codons in all three reading frames [6]. The mice were backcrossed extensively with the C57BL/6 strain. The homozygous mice were born at a lower than expected Mendelian frequency (9%), and were smaller than their littermates initially [6], but caught up in size around 2 months of age and were reproductive. The mice were maintained and bred at the Animal Resource Center at University of Texas Medical Branch. All possible steps were taken to avoid animals from suffering as well as keeping the number of animals used in the studies to a minimum. All of the experiments and procedures were approved by the Institutional Animal Care and Use Committee at University of Texas Medical Branch.

### Auditory function testing

For auditory function testing, the mice were anesthetized by intraperitoneal injection of pentobarbital (60 mg/kg body mass). The mice were kept on a heating pad for body temperature control during all auditory tests. The auditory tests were performed in a sound proof environment. Wild type (*Casp3*^+/+^), heterozygous (*Casp3*^+/-^) and homozygous (*Casp3*^-/-^) mice at 3 months of age were tested, and the results were compared among different genotypes.

For ABR, subcutaneous needle electrodes were placed adjacent to the left and right pinna and at the occipital region. Sound stimuli were delivered to the ear by an ear probe. ABR recordings were performed using Nicolet CA2000 system and software (Cardinal Health, Madison, WI, U.S.A.). Averages of 500 responses to click stimuli were recorded for descending 5 dB stimulus steps to determine the threshold. Averages of the better-hearing ear were compared among the different genotypes.

For DPOAE, tests were performed using the Starkey DP2000 system and software (Starkey Laboratories, Eden Prairie, MN, U.S.A.). Signal-to-noise ratio of 2F1-F2 were obtained and analyzed at various F2 values from 0.5- to 16-kHz. F1/F2 = 1.22, F1 = 65dB, F2 = 55dB.

### Vestibular function testing

For vestibulo-ocular reflex (VOR) testing, we used a custom-made rodent centrifuge [25], modified for use in mice. Details are described [25], except for modifications as follows. After brief isoflurane inhalation anesthesia, the mouse body is restrained with a plastic cone and then secured onto a custom-made bed. The anterior portion of the head including the pinna is placed outside of the narrow end of the cone. Then the nose and maxilla is secured with a bite block, with the head restrained with a custom-made adjustable head-mount. The nose is angled in a 35° nose down position, resulting in an earth horizontal plane for stimulation of the lateral semicircular canal. Forearms are restrained inside the plastic cone, and hind legs are extended, restrained, and secured with a custom-made leg restraint. Bite block and bed are attached to a mini-platform that is designed to dock into the centrifuge to assure stability of location. After securing the mouse into the centrifuge, the mouse was kept quiet for 10-15 minutes to assure full awakening and to stabilize before the test is started. Mice were tested at 3 months of age. Eyes were treated with 1% pilocarpine to restrict pupils. Horizontal eye position was derived from tracking the pupil center using a least squares fit to a circular disk model [26]. Calibration scale factors were geometrically derived based on the ocular globe image size, and verified by rotating a camera about the midpoint of the globe. Following differentiation and fast phase removal, nonlinear least squares sinusoidal curve fits to the remaining slow components of horizontal eye velocity were used to determine the gain and phase of the horizontal canal VOR over at least three successive cycles. The frequency response of the VOR in darkness was characterized during horizontal rotation at five frequencies ranging from 0.05 Hz to 0.8 Hz, with peak velocity of 60 deg/sec. The linearity of the VOR was tested at 0.2 Hz using peak velocities ranging from 60 to 120 deg/sec.

### Anatomical studies of the inner ear

The mice were euthanized with CO_2 _inhalation followed by decapitation following the IACUC protocol. The inner ears were dissected from the temporal bones, and were fixed with 4% paraformaldehyde in phosphate buffered saline (PBS) for more than 2 hours. The surrounding soft tissue and mastoid air cells were carefully cleaned using fine forceps. Inner ears from mice were collected at different ages up to 6 months of age.

### Whole mount preparation of the inner ear

Mice aged 6 months or younger were used. The mice were sacrificed with CO_2 _inhalation followed by decapitation. The temporal bone was immediately dissected and perfused with 4% paraformaldehyde in PBS for 1 hour. Then the organ of Corti and vestibular sensory epithelia were carefully dissected. The specimen were washed with PBS for 5 minutes, permeabilized with 0.5% Triton X-100 for 30 minutes, and again washed with PBS for 5 minutes x3. Then, under dark conditions, the specimen was incubated with Alexa fluor 568-conjugated Phalloidin diluted in PBS for 60 minutes, and then was incubated with Hoechst33342 diluted in PBS for 15 minutes to stain the hair cell stereocilia and nuclei, respectively. After washing with PBS for 5 minutes x3, the specimen was mounted with Slowfade Gold antifade mounting solution (Invitrogen) and sealed. The inner ear sensory epithelia were observed under fluorescent microscopy (Nikon TE2000) at 100x, 150x, 400x, 1000x, and 1500x magnifications. Images were captured using Nikon NIS element software (Nikon).

Hair cell count: Cells with stereocilia labelled with Alexa fluor 568 phalloidin at the apical surface were counted. A combination of 150x and 400x magnifications were used to extract individual cells as accurate as possible for counting.

### Statistical analysis

Results from the auditory and vestibular testing were compared among *Casp3*^*+/+*^*, Casp3*^*+/- *^and *Casp3*^*-/- *^mice. Statistical difference (*P *< 0.05) was assessed by ANOVA, or Student's *t *test using the StatistiXL version 1.6 (available from http://www.statistixl.com/) with the Windows™ version of Microsoft Excel™.

## Authors' contributions

TM designed all of the experiments, carried out pilot studies, supervised all experiments, and drafted the manuscript. PA carried out the VOR experiments, and data analysis. LW carried out anatomical studies, auditory studies and VOR studies, and data analysis. ER carried out VOR studies. SW modified the VOR software, carried out VOR analysis, supervised analysis and interpretation of VOR data, and was involved significantly in revising the manuscript. AP was involved significantly in revising the manuscript related to vestibular functional studies. MW offered critical and valuable knowledge and discussions related to *Casp3 *knockout mice, and was involved in revising the manuscript. RR carried out anatomical studies and data analysis of the mice. All authors read and approved the final manuscript.
